# An Efficient Method of Hepatoduodenal Ligament Taping for Pringle’s Maneuver During Robotic Liver Resection

**DOI:** 10.7759/cureus.39214

**Published:** 2023-05-19

**Authors:** Yusuke Uemoto, Takahisa Fujikawa, Taisuke Matsuoka

**Affiliations:** 1 Surgery, Kokura Memorial Hospital, Kitakyushu, JPN

**Keywords:** anatomical liver resection, extracorporeal tourniquet method, hepatoduodenal ligament, pringle's maneuver, robotic liver resection

## Abstract

Background and objective

Pringle’s maneuver is often applied to reduce bleeding during liver resection (LR), although the taping of the hepatoduodenal ligament (HL) is challenging and dangerous due to the lack of tactile perception in robotic liver resection (RLR). In this study, we describe a secure and easy HL taping method in RLR.

Methods

Twenty-seven cases that underwent RLR at our institution from April to November 2022 were examined. For the HL taping, a taping tool was prepared with a flexible catheter and 3 mm-thick silicon tape. The lesser omentum was opened, the taping tool was inserted behind the HL, and the HL was encircled by silicon tape. The length of time required for taping and the number of attempts were measured. Intraoperative blood loss, the occurrence of post-hepatectomy liver failure (PHLF), and complications were examined.

Results

A total of 18 cases were analyzed, after excluding cases in which taping was not attempted due to adhesion from repeated hepatectomy. The median time taken for taping was 55 seconds (range: 11-162 seconds), and the median number of attempts for taping was one (range: 1-4). No accidental injury was observed during the procedure. Intraoperative blood loss was 24 mL (range: 5-400). No PHLF occurred, and complications occurred in two cases (one case of bile leakage and one case of pulmonary atelectasis).

Conclusion

Based on our findings, our method enables secure and time-efficient HL taping in RLR.

## Introduction

Liver resection (LR) is a successful method of treatment for colorectal cancer metastases and hepatocellular carcinoma. Several strategies are used to control bleeding during LR as this procedure tends to induce a lot of bleeding [1]. Pringle's maneuver, which involves applying a tourniquet to the hepatoduodenal ligament (HL) to block the hepatic inflow, is one of the most efficient ways to reduce bleeding during LR [2].

The tourniquet of HL has often been performed in open, laparoscopic, and robotic LR (RLR). The taping of the HL can be done relatively easily in open LR. Several studies have been published on Pringle’s maneuver in laparoscopic LR. However, the laparoscopic LR method cannot be directly applied to RLR due to the different trocar placement between laparoscopic LR and RLR. In addition, to prevent accidental injury to the HL, a secure HL taping method is necessary because robotic forceps lack tactile perception. In this paper, a secure and time-efficient HL taping method in RLR is demonstrated.

## Materials and methods

From April to November 2022, 27 cases underwent RLR at the Kokura Memorial Hospital. All these cases were scheduled for RLR and were evaluated with CT, blood tests, and an indocyanine green retention test before surgery. Informed consent was obtained preoperatively in each case. The Kokura Memorial Hospital Clinical Research Ethics Committee approved the study protocol (#21021002), which complied with the Declaration of Helsinki.

Measurements were made of the length of time required for taping and the number of attempts. Intraoperative blood loss, the occurrence of post-hepatectomy liver failure (PHLF), and postoperative complications were examined.

Surgical technique

The Da Vinci Xi surgical system (Intuitive Surgical, Inc., Sunnyvale, CA) was used for RLR [3]. Generally, the procedure is performed by using four trocars for the robotics and one 12-mm trocar for the assistant surgeon.

**Figure 1:**
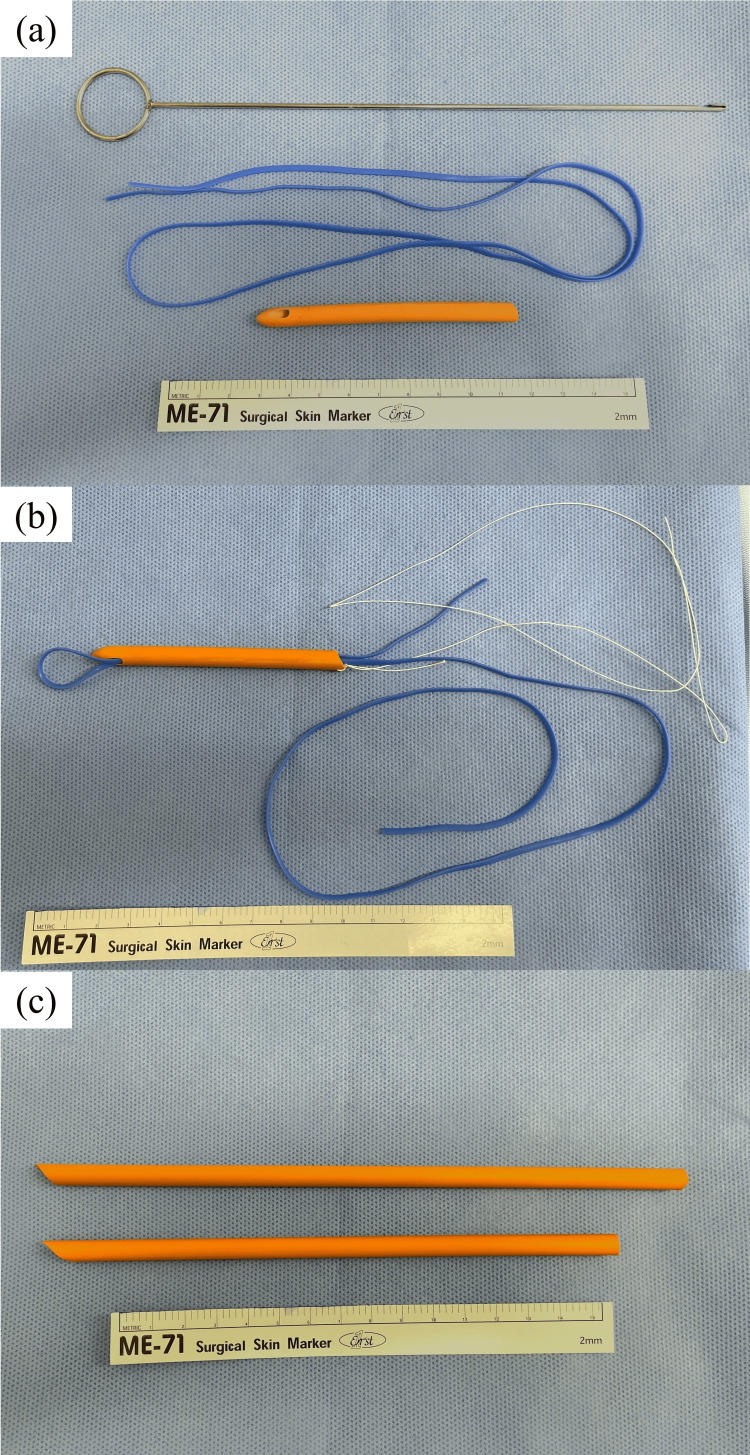
Preparation of the taping tool with a catheter and silicon tape (a) The silicon tape was 3 mm in thickness and 76 cm in length, and the catheter was cut to 8.5 cm in length. (b) The tape was inserted into the catheter with a loop at the tip, and a traction string was fastened to the tail side of the catheter. (c) The tourniquet catheter was cut at an angle. Catheters with 21-23 cm in length were used according to the distance from the hepatoduodenal ligament to the abdominal wall

Concerning the device for HL taping and the procedure for tourniquet of HL, Okuda et al. have previously reported the method of tourniquet of HL for laparoscopic LR [4], and we modified this method for RLR. A taping tool was prepared with a 7 mm-diameter catheter (Nelaton catheter, Izumo Health Co., Nagano, Japan) and a relatively thick and long silicon tape (3 mm in thickness and 76 cm in length; Gadelius Medical Inc., Tokyo, Japan) (Figure 1). The catheter was cut to 8.5 cm in length, and the tape was placed through the catheter. A traction string was fastened to the tail side of the catheter.

**Figure 2:**
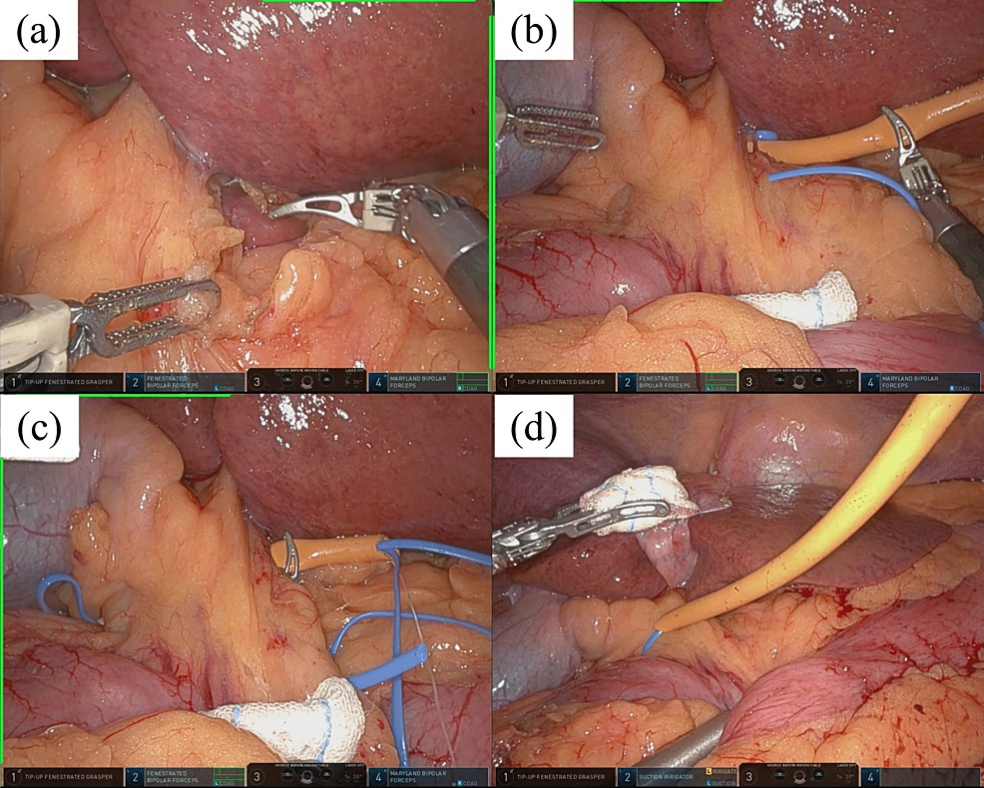
The taping procedure for HL in RLR (a) The lesser omentum was opened at the left side of the HL. (b) The taping tool was inserted from the opened lesser omentum and passed behind the HL. (c) The tape on the tip of the catheter was caught on the right side of the HL. (d) The catheter to perform a tourniquet with the taping of HL was inserted through the abdominal wall HL: hepatoduodenal ligament; RLR: robotic liver resection

The HL taping procedure is described in Figure 2 and Video 1. The taping tool was delivered into the abdominal cavity through the 12-mm trocar. First, to straighten the HL, the duodenum was pulled caudally with the robotic forceps, and the hepatic round ligament was elevated ventrally with the assistant's forceps. Second, the tool was inserted from the opened lesser omentum and passed behind the HL. The taping tool was carefully advanced while probing with the multi-joint robotic forceps. The tape on the tip of the catheter was caught on the contralateral side, and the taping of the HL was performed. The catheter was removed by pulling the string from the 12-mm trocar. The tape attached to HL was placed in a tourniquet catheter (21-23 cm in length) through the abdominal wall. The tourniquet catheter was positioned on the patient’s left side if the lesion was in the right lobe, and on the right side if the lesion was in the left lobe. When blocking the hepatic inflow, the tape was pulled from outside the body and the tourniquet was applied (Video 1).

**Video 1:** The procedure for the HL taping using the taping tool described in the current paper HL: hepatoduodenal ligament

## Results

The taping of HL was performed in 19 cases. Adhesions due to a repeated hepatectomy were the most common reason for not attempting taping (five cases). There were two cases in which HL taping was not possible because of adhesions due to gastrectomy and cholecystectomy. In one case, HL taping was not performed at the discretion of the operator due to a small partial resection of the liver surface. Of the 19 cases, 18 cases were analyzed, excluding one in which the taping was performed laparoscopically. Patient characteristics and surgical procedures are shown in Table 1. The median time taken for taping was 55 seconds (11-162) and the median number of attempts for taping was one (1-4) (Table 2). The patient in whom HL taping was attempted four times (in 162 seconds) was extremely obese (body mass index of 38). The HL was fatty and extremely thick. The robotic forceps' grip angle and the degree of HL straightening were adjusted to complete the HL taping procedure. No accidental injury was observed during the procedure. Intraoperative blood loss was 24 mL (5-400). No incidence of PHLF occurred, and postoperative complications occurred in two cases: one case of bile leakage [Grade A by International Study Group of Liver Surgery (ISGLS)] [5] and one case of pulmonary atelectasis. The mortality rate was zero.

**Table 1:** Patient characteristics and surgical procedures HCC: hepatocellular carcinoma; ICG R15: indocyanine green retention rate at 15 minutes

Variables	Values
Male gender, n (%)	14 (78%)
Age, years, median (range)	75 (46-83)
Disease	
HCC, n (%)	9 (50%)
Colorectal cancer liver metastases, n (%)	8 (44%)
Benign tumors, n (%)	1 (6%)
Type of resection	
Anatomical resection, n (%)	10 (56%)
Non-anatomical resection, n (%)	8 (44%)
Child-Pugh classification A, n (%)	18 (100%)
ICG R15, median (range)	13.3 (4.4-83)
Repeated hepatectomy, n (%)	3 (17%)

**Table 2:** HL taping procedure and perioperative outcomes in the current study HL: hepatoduodenal ligament; PHLF: post-hepatectomy liver failure; LOS: length of stay

Variables	Values
Time taken for taping, seconds, median (range)	55 (11-162)
Number of attempts for taping, median (range)	1 (1-4)
Number of Pringle’s maneuvers performed, median (range)	1 (0-12)
Blood loss, ml, median (range)	24 (5-400)
Operation time, minutes, median (range)	317 (130-568)
Transfusion	0
PHLF	0
Postoperative complications, n (%)	2 (11%)
LOS, days, median (range)	8 (5-25)

## Discussion

In this study, a secure, easy, and time-efficient HL taping method in RLR is described. With a simple HL taping tool involving the silicon catheter and tape, the HL taping during RLR was securely and time-efficiently performed without any accidental injury to the liver or the HL. We recommend that this method be one of the preferred techniques for HL taping in RLR.

The HL taping in the laparoscopic LR is typically performed by inserting laparoscopic forceps into the dorsal side of the HL according to tactile and visual cues. It is challenging to properly insert forceps through the dorsal side of the HL at the location of the trocar in the RLR due to the difference in trocar location between the laparoscopic LR and RLR. For example, an additional trocar must be needed in the right flank to insert the laparoscopic forceps dorsally to the HL in RLR [6]. Although there have been some previous reports about Pringle’s maneuver in RLR [7,8], blind operations with robotic forceps through the dorsal side of the HL carry the risk of accidental injury due to the absence of touch. HL dorsal injuries can cause serious accidents. On the other hand, our HL taping method, which took advantage of a catheter with a soft tip and a multi-joint function of a robotic arm, was found to be safe and practical.

The method that Okuda et al. described used a tetron tape [4], whereas, in our method, a silicon tape is used. The silicon tape has less frictional resistance with the catheter than the tetron tape, making it possible to perform and release the tourniquet smoothly. The usually used silicon tape during LR for encircling the vessels is generally thin (1-2 mm in thickness) and short (40-45 cm in length). On the other hand, the silicon tape used in the current method is thick and long (3 mm in thickness and 76 cm in length), which does not break even when stretched strongly and thus can be securely used even in the extracorporeal tourniquet system in RLR. According to some reports, different tapes have been used for Pringle's maneuver [9-11], but it is still unclear as to which tape is optimal. However, at least for the procedures in RLR, we believe that the use of more flexible and easy-to-use thick silicon tape is one of the most efficient options.

Although both intra and extracorporeal Pringle’s maneuvers have been described in the past, the extracorporeal method has many advantages, such as a sufficient tourniquet and easy operation [8]. Because the incision for the extracorporeal Pringle’s maneuver is small (approximately 5 mm), it is thought that it leads to few problems in terms of pain and appearance.

It was assumed that adhesions around the HL brought on by repeated hepatectomy were the main reason why taping of the HL was not feasible. In this cohort, adhesion barriers were applied in every instance where HL taping was feasible after repeated hepatectomy. The number of repeated hepatectomy procedures has increased due to the improvement in image diagnosis and the development of multidisciplinary treatment, and hence it is speculated that the use of adhesion barriers around HL might be effective. In addition, even if dissection of adhesions is required, it is expected that the magnifying and stereoscopic effect and precise operation of dissection in robotic surgery will enable safer HL taping, but this has to be confirmed through future investigations.

## Conclusions

Our findings reveal that our HL taping method is secure and practical in RLR. With a simple HL taping tool involving the silicon catheter and tape, the HL taping during RLR was securely and time-efficiently performed without any accidental injury to the liver or the HL. We recommend that this method be chosen as one of the preferred techniques for HL taping in RLR.
